# Characterisation of baseline microbiological and host factors in an inception cohort of people with surgical wounds healing by secondary intention reveals circulating IL-6 levels as a potential predictive biomarker of healing

**DOI:** 10.12688/wellcomeopenres.15688.2

**Published:** 2020-11-19

**Authors:** Hannah Buckley, Jo Dumville, Michael Hodgkinson, Debbie Wearmouth, Gavin Barlow, Marjan van der Woude, Nicky Cullum, Ian Chetter, Dimitris Lagos

**Affiliations:** 1 York Trials Unit, Department of Health Sciences, University of York, York, YO10 5DD, UK; 2 Division of Nursing, Midwifery and Social Work, School of Health Sciences, Faculty of Biology, Medicine and Health, University of Manchester, Manchester, M13 9NT, UK; 3 York Biomedical Research Institute, Hull York Medical School, University of York, York, YO10 5DD, UK; 4 Department of Infection, Hull and East Yorkshire Hospitals NHS Trust, Hull, HU3 2JZ, UK; 5 Research and Innovation Division, Central Manchester University Hospitals NHS Foundation Trust, Manchester, M13 9PL, UK; 6 Academic Vascular Surgical Unit, Hull York Medical School / Hull University Teaching Hospital NHS Trust, Hull, HU3 2JZ, UK

**Keywords:** Surgical wounds, biomarkers, IL-6, microbiology, bacteria, healing

## Abstract

**Background:** More than 2 million people per year are treated for surgical wounds in the UK.  Over a quarter of these wounds are estimated to heal by secondary intention (from the “bottom up”) resulting in further complications and requiring increased healthcare resources. Identification of microbiological or host biomarkers that can predict healing outcomes may help to optimize the management of surgical wounds healing by secondary intention. However, the microbial and host factor heterogeneity amongst this diverse population is completely unexplored.

**Methods:** We demonstrate feasibility of determining presence and levels of wound microbes and systemic host factors in an inception cohort of 54 people presenting with surgical wounds healing by secondary intention, who were subsequently followed-up for a period of 12-21 months. We present descriptive statistics for plasma levels of inflammatory, angiogenic cytokines and microRNAs, and we identify a range of wound colonizing microbes. We tentatively explore association with healing aiming to generate hypotheses for future research.

**Results:** We report a potential correlation between poor healing outcomes and elevated interleukin (IL)-6 plasma levels at presentation (ρ=0.13) which requires confirmation.

**Conclusions: **This study demonstrates the degree of biological heterogeneity amongst people with surgical wounds healing by secondary intention and proves the feasibility of embedding a biomarker discovery study in a cohort study in surgical wounds. Our results are essential for designing large biomarker discovery studies to further investigate the potential validity of circulating IL-6 or other factors as novel predictive biomarkers of healing for surgical wounds healing by secondary intention.

## Introduction

Over ten million surgical operations are performed in the United Kingdom (UK) each year ^1^. Surgical wounds are usually closed by primary intention, where wound edges are held together whilst healing occurs using sutures (stitches), staples, adhesive or clips. However, some wounds heal by secondary intention, where the wound is left to open to heal from the ‘bottom up’. This type of healing may occur when healing by primary intention fails (for example, due to infection) or may be planned (for example, following excision of a pilonidal sinus, or incision and drainage of a perianal abscess). Wounds healing by secondary intention may be subsequently closed surgically.

Two published audit studies from the North of England estimated that surgical wounds healing by secondary intention (SWHSI) constitute approximately 28% of all prevalent acute (mainly surgical/traumatic) wounds that were receiving wound care provision ^2, 3^. More recent research, also in the North of England, estimated that SWHSI have a point prevalence of 4.1 per 10,000 population (95% confidence interval (CI) = 3.5 to 4.7) ^4^.

SWHSI are managed in both acute and community settings where they can be challenging to manage as they present specific management problems such as high levels of exudate and a risk of cross-infection and trauma. In our cohort study, we estimated that SWHSI have a median time to healing of 86 days (95% CI: 75 to 103) ^5^ and therefore whilst accurate cost data are not available, SWHSI are clearly costly for the National Health Service (NHS). Importantly, we have also shown they have a huge and adverse impact on people’s quality of life ^6^. SWHSI are clearly a topic deserving of more research and effective treatments.

The identification of prognostic factors that predict healing, complications and/or treatment response in people with SWHSI would enable a paradigm shift towards improving wound management and reducing healthcare costs. However, there is a notable lack of research on the immunological and microbiological phenotype of SWHSI. Although some research on the predictive biomarkers of healing has been undertaken in wounds such as venous and diabetic ulcers ^7, 8^, there are no studies on biological biomarkers of SWHSI healing. A potential challenge in identifying predictive biomarkers in SWHSI is the heterogeneity of the patients (including their underlying pathophysiology) and the wounds.

Here, we present an initial characterisation of the immunological and microbial wound landscape in a small cohort of patients with SWHSI. We demonstrate that a biomarker discovery study can be embedded in research in this field, leading the way for future research. We report a wide range of microbial species present in SWHSI and determine circulating levels of selected cytokines and nucleic acids (microRNAs) at presentation. We chose to determine levels of interleukin (IL)-6 and angiopoietin-2 (ANG-2), two cytokines that are have been suggested to be associated with wound inflammation, angiogenesis, and healing ^9– 12^. Similarly, we determined plasma levels of miR-146a and miR-126, which are also implicated in skin inflammation, angiogenesis, and wound healing ^13, 14^. We provide exploratory information on heterogeneity in wound and systemic parameters across individuals with SWHSI, which can be used for designing larger biomarker discovery studies. Furthermore, we cautiously investigate possible associations between baseline microbiological and immunological factors and wound healing, and identify blood plasma IL-6 levels as a potential candidate biomarker of healing of SWHSI.

## Methods

### Study participants

This feasibility study was nested in a larger inception cohort study ^5^ that recruited participants with an incident SWHSI (present for 3 weeks or less at recruitment into the cohort). These wounds were defined as ‘open surgical wounds healing from the bottom up via the formation of granulation tissue’ and where the open wound required treatment. Our definition of SWHSI inclusion and exclusion criteria are detailed in Table 1.

**Table 1.** Definition of surgical wounds healing by secondary intention (SWHSI) used in our study.

Included
1. Wounds that had been left open following surgery (i.e. due to contamination, swelling or infection or because there is an empty space below the wound)
2. Wounds that may have been closed by sutures, clips etc. at surgery but then partially or completely opened, or dehisced
3. Open wounds resulting from surgical or sharp debridement which may have been non-surgical in origin e.g. surgical debridement of a grade III/IV pressure ulcer, sharp debridement of a foot ulcer
Excluded
1. Wounds with planned delayed primary closure (i.e. surgical wounds left open for 5 to 7 days after surgery with planned closure thereafter). However, if these wounds then became SWHSI (as defined above) they became eligible for inclusion
2. Wounds left open with no planned healing, e.g. stoma, tracheotomies, gastrostomies
3. Surgery without an incision on the skin surface, e.g. tonsillectomy, dilation and curettage (i.e. “internal” wounds)
4. Split-skin donor graft sites
5. Nail avulsions
6. Cavities resulting from dental extractions
7. Operations involving the eye (i.e. cataract surgery and removal of the eye)
8. Wounds that are a consequence of minor dermatological or plastic surgery (e.g. removal of warts, skin tags) or diagnostic procedures (e.g. punch biopsy)
9. Recurrence of a SWHSI that had previously healed
10. Participants previously recruited to this study

No formal sample size calculation was conducted as this was a feasibility study, the achieved sample size of 54 participants was deemed sufficient to provide initial descriptive statistics and identify potential associations between tested biological and epidemiological factors for healing. 

We collected baseline demographic and wound characteristics data including: participant age, gender, co-morbidities and smoking status. Participants were then followed for between 12 months and 21 months to determine time to wound healing and data were collected on key events such as re-operations and infections. All participants provided written informed consent to participate in the study. The study was conducted according to the principles of the Declaration of Helsinki 1975 and ethics approval was granted by National Research Ethics Service Committee Yorkshire and the Humber South Yorkshire on 06.12.2012 (Reference: 12/YH/0537). 

### Biological sample collection, transport and storage

At entry into the cohort study some participants gave consent to provide up to two biological samples (a wound swab and a blood sample). Research nurses obtained wound swabs at baseline using a standardised technique: the wound was not cleansed before taking the swab to ensure the maximum number of bacteria were present and the technique aimed to swab purulent discharge if it was present. In the absence of purulent discharge, the swab was taken from an area of the wound containing viable tissue where possible (e.g. granulating tissue). To collect the specimen the wound swab was rotated over a 1cm ^2^ area of the wound, applying light pressure with the aim of saturating the swab. If the wound was dry, the swab was moistened with sterile water or sterile saline to aid in the collection of the specimen. Research nurses were asked to avoid taking the sample from areas of the wound covered with slough or necrotic tissue. All swabs were then transported in bacteriological transport medium, at room temperature, within three hours of collection by courier.

Blood samples were collected from consenting patients at baseline using standard venepuncture techniques. Samples were transported within three hours to the University of York and logged in for plasma isolation following standard operating procedures. In brief, samples were centrifuged and the cellular component of the blood sample discarded. The remaining plasma sample was aliquoted (at least 7 aliquots of 0.5ml per participant) and stored at below -70 degrees centigrade. All practice followed good laboratory practice guidelines.

### Microbiological analysis

Basic microbiological analyses were conducted using local laboratory standard operating procedures based on the UK Standards for Microbiological Investigations produced by Public Health England ^15^. Wound swabs were inoculated onto the following agar plates sequentially in order to obtain single bacterial colonies: Blood agar (mainly for Gram-positive bacteria); cystine-lactose-electrolyte-deficient (CLED) agar (mainly for Gram-negative bacteria) and anaerobic blood agar (for anaerobic bacteria). Blood agar plates were incubated in CO _2 _at 37°C for 24 hours then read and re-incubated for a further 24 hours; CLED plates in an aerobic atmosphere at 37°C for 24 hours; and anaerobic agar plates in an anaerobic atmosphere at 37°C for 48 hours then read and re-incubated as necessary for a further 5 days with a 5μg metronidazole disc placed at the time of first streaking out from the main inoculum.

Subsequent microbial growth was differentiated and identified according to the UK Standards for Microbiological Investigations produced by Public Health England ^15^ and qualitatively categorized into the following groups: 1) no bacterial growth; 2) the presence of skin commensals only (coagulase negative *Staphylococcus* species, *Micrococcus* species, and diphtheroid bacilli); and 3) growth of one or more of the following potentially pathogenic bacteria (with or without the presence of skin commensals): *Staphylococcus aureus* (methicillin sensitive and resistant), Lancefield Group A, B, C and G Streptococci, anaerobes, coliforms and *Pseudomonas* spp.

***Measurement of plasma cytokine levels and circulating microRNA levels.*** IL-6 and ANG-2 levels were measured with commercially available enzyme immunoassays (R&D Systems: Human IL-6 Quantikine ELISA Kit, product number: D6050; Human Angiopoietin-2 Quantikine ELISA Kit, product number: DANG20), following manufacturer’s instructions, using the plasma samples following blood separation.

Two microRNAs were measured, miR-146a and miR-126. Ribonucleic acid (RNA) was extracted from 50µl of plasma. MiRNA reverse transcription was performed (TaqMan MicroRNA Reverse Transcription Kit; Applied Biosystems), as per manufacturer’s instructions, and relative levels were determined by quantitative polymerase chain reaction (PCR) using Taqman microRNA primers (Applied Biosystems). TaqMan Assays (including primers) for miR-146a and miR-126 were purchased from ThermoFischer (catalogue number 4440886 for both). Master mixes were prepared in Taqman Universal PCR Master Mix (catalogue number 4304437). PCR was carried out using a StepOnePlus Real Time PCR System with the following cycling conditions: 95°C for 10mins, 40 cycles of [95°C for 15 seconds, 60°C for 30 seconds, 72°C for 40 seconds], 95°C for 60 seconds. The DeltadeltaCt method was used for quantification. cDNA (corresponding to 1ng total RNA) from primary dermal microvascular endothelial cells was used as reference.

### Data analysis

Data analysis was undertaken using Stata v13 or later ^16^, proportions of missing data were explored for all variables. Population characteristics are presented descriptively. Wound healing is summarised using a Kaplan-Meier curve and median time to healing is presented with 95% CI. Baseline immunological parameter concentrations are presented descriptively both overall and stratified by healing status at the end of the main cohort follow-up period. Where observed values were below the assay of the test, a value of half the assay was imputed for analysis purposes. Due to the skewed nature of the concentration variables, t-tests were performed on logarithmically transformed data to assess the difference in means between participants who healed and those who did not. Pearson’s correlation coefficients examined association between time to healing and parameter concentrations.

Microbiological species are illustrated using frequencies and percentages for the sample overall and by healing status. Fisher’s exact tests were used to examine possible associations between healing status (healed/not) and the presence of the three most common microbes states in the wound at baseline: gastrointestinal bacteria (defined as the presence of one or more of coliform ( *Enterobacteriaceae, Pseudomonas* or *Enterococcus* species), coliforms and skin commensals. Log-rank tests compared time to healing by presence of microbiological parameters. T-tests on log-transformed data compared mean concentrations of immunological parameters by presence of selected microbiological parameters. Microbiological parameters to be examined were selected *a priori* to minimise the risk of Type I error.

Given the limited sample size, detailed adjusted analysis was not possible, however we identified diabetes as a potential confounding factor of interest due to the well-established link between diabetes and impaired healing ^17, 18^, and studies indicating deregulated levels of IL-6 ^19, 20^, ANG-2 ^21, 22^, miR-146a ^23, 24^, and miR-126 ^25, 26^ in individuals with diabetes. Thus we also present analysis of immunological factors by diabetes status. For microbial analysis we only present unadjusted analyses.

## Results

### Description of study population

In total, 54 (13.7%) people within the overall cohort of 393 eligible individuals consented to this sub-study of whom 51 (94.4%) provided a blood sample, and 44 (81.5%) a wound swab, with 42 (78%) provided both. Table 2 shows baseline characteristics of these consenting individuals; mean age was approximately 52 years (SD 19.1) and 61.1% of participants were male. Non-smokers constituted 64.8% of the sample and 27.8% were current smokers (or had quit in the last year). Cardiovascular disease (33.3%) and diabetes (25.9%) were common co-morbidities. Baseline wound infection was present for 12 participants (22.2%); intravenous antibiotics for SWHSI were reported for 12 participants (22.2%) and oral antibiotics for 10 participants (18.5%).

**Table 2.** Summary statistics for participant baseline characteristics. SWHSI: surgical wounds healing by secondary intention.

		N=54
**Gender**	Male, n (%)	33 (61.1)
**Age in years**	Mean (SD)	51.7 (19.1)
	Median (range)	52.3 (19.5, 83.2)
**Smoking status**	None in the last 10 years, n (%)	35 (64.8)
	None current but in last 10 years, n (%)	4 (7.4)
	Current or quit in last year, n (%)	15 (27.8)
**Co-morbidity**	Cardiovascular disease, n (%)	18 (33.3)
	Diabetes, n (%)	14 (25.9)
	Peripheral vascular disease, n (%)	9 (16.7)
	Cancer, n (%)	7 (13.0)
	Arthritis, n (%)	6 (11.1)
	Orthopaedics, n (%)	6 (11.1)
	Airways, n (%)	5 (9.3)
	Stroke, n (%)	5 (9.3)
	Other, n (%)	3 (5.6)
**Number of** **SWHSI**	Mean (SD)	1.1 (0.32)
	Median (range)	1 (1, 2)
**Wound infection,**	Yes *, n (%)	12 (22.2)
**Antibiotic use ^¶^**	Yes ^§^, n (%)	22 (40.1)

* Baseline wound infection missing for n=2 (3.7%); ¶ SWHSI related antibiotic use § Specifically: oral (n=10, 18.5%); intravenous (n=12, 22.2%); and no topical antibiotic use.

Baseline wound characteristics are shown in Table 3. Over 60% of SWHSI were planned; one-quarter because it was not possible to approximate the wound edges; one-third due to infection; and a further 37% of wounds partially dehisced after surgery. Mean baseline SWHSI area was 36.0cm ^2 ^(SD 68.8). Colorectal and vascular surgery accounted for over 60% of surgeries (38.9% and 22.2% respectively); with nearly 60% of wounds being abdominal or leg wounds (33.3% and 24.1% respectively).

**Table 3.** Summary statistics for baseline wound characteristics. SWHSI: surgical wounds healing by secondary intention.

		N=54
**SWHSI area (cm ^2^)**	Mean (SD)	36.0 (68.8)
	Median (range)	7.6 (0.1, 384)
**Reason for SWHSI**	Dehisced, n (%)	1 (1.9)
	Partially dehisced, n (%)	20 (37.0)
	Planned due to wound edges, n (%)	14 (25.9)
	Planned due to infection, n (%)	18 (33.3)
	Planned for other reason, n (%)	1 (1.9)
**Surgery leading to SWHSI**	Colorectal, n (%)	21 (38.9)
	Vascular, n (%)	12 (22.2)
	Other, n (%)	11 (20.4)
	Plastics, n (%)	5 (9.3)
	Obs/gynae, n (%)	3 (5.6)
	Orthopaedic, n (%)	2 (3.7)
Location of SWHSI *	Abdomen, n (%)	18 (33.3)
	Foot, n (%)	7 (13.0)
	Leg, n (%)	13 (24.1)
	Peri-anal area, n (%)	7 (13.0)
	Other, n (%)	5 (9.3)

*4 participants missing information on location of SWHSI (7.4%).

### Wound healing

**Figure 1.**
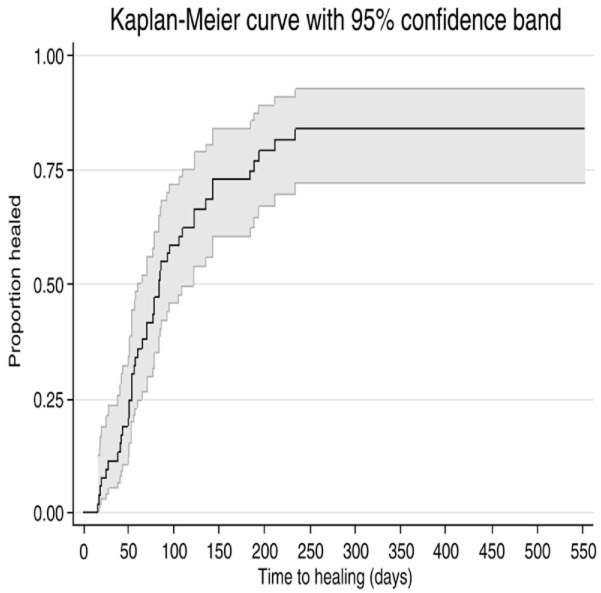
Kaplan-Meier curve of time to wound healing.

Healing occurred in 43 participants during follow-up (79.6%) and 11 individuals (20.4%) had a recurrence within the study period. The median time to healing in this population was 83 days (95% CI: 59 to 121 days; Figure 1) as compared with 86 days (95% CI: 75 to 103) in the larger cohort ^5^.

***Analysis of plasma IL-6, ANG-2, miR-146a, and miR-126 levels in SWHSI.*** Table 4 shows summary statistics for baseline IL-6 and ANG-2 concentration. Mean ANG-2 concentration in non-healers was nearly twice that of healers (5876pg/ml compared with 3412pg/ml) although this difference was not statistically significant at the 5% level (p=0.06). The mean baseline IL-6 concentration was significantly higher for those who did not heal than that for those who healed (p<0.01) with mean values of 86.9pg/ml (SD 104.4pg/ml) and 29.8pg/ml (SD 136.9pg/ml) respectively. In general, people with higher levels of ANG-2 took longer to heal, although this positive correlation was weak (ρ=0.35). There was also a very weak positive correlation between time to healing and IL-6 concentration suggesting that those with higher levels of IL-6 may take longer to heal (ρ=0.13). 

**Table 4.** Summary statistics for baseline immunological parameter concentrations, overall and by healing status.

	Overall N=51 *	Healed N=42	Not healed N=9	Difference ^‡^ (95% CI)	p-value ^§^
*Angiopoietin-2 (pg/ml)*					
Mean (SD)	3847 (2998)	3412 (1501)	5876 (6247)	1.4 (1.0, 2.0)	0.06
Median	3319	3313	4781	-	-
Min, max	1473, 22195	1473, 7905	2097, 22195	-	-
*Interleukin-6 ^†^ (pg/ml)*					
Mean (SD)	39.8 (132.6)	29.8 (136.9)	86.9 (104.4)	**5.2 (1.9, 14.5)**	**<0.01**
Median	7.3	5.6	64.5	-	-
Min, max	1.6, 893.1	1.6, 893.1	1.6, 300.4	-	-
*miR-126 (AU/ml)*					
Mean (SD)	0.5 (0.4)	0.5 (0.4)	0.6 (0.4)	1.1 (0.7, 1.8)	0.74
Median	0.4	0.4	0.4	-	-
Min, max	0.1, 2.7	0.1, 2.7	0.2, 1.3	-	-
*miR-146a (AU/ml)*					
Mean (SD)	9.3 (6.9)	9.5 (7.2)	8.5 (5.1)	0.93 (0.5, 1.7)	0.82
Median	7.9	7.6	9.6	-	-
Min, max	1.1, 26.8	1.1, 26.8	1.3, 15.8	-	-

*3 individuals without a blood sample; ‡ difference in geometric means, presented in terms of geometric means due to log-transformation of data prior to testing.; § log rank test p-value; † 14 samples (13 healed; 1 unhealed) where concentration was below the assay detection limit of 3.1pg/ml with values set at 1.55 pg/ml (half the assay).

The mean of miR-126 was 0.5AU/ml (SD 0.4AU/ml) and for miR-146a was 9.3AU/ml (SD 6.9AU/ml). There was no evidence of a difference between healed and unhealed participants in terms of mean miR126 (p=0.74) or miR146a (p=0.82). There was a negligible association between time to healing and miR-126 (ρ=0.03) and miR-146a (ρ=-0.04).

***Healing and diabetes.*** Time to healing was explored in a log-rank test by diabetic status (14 participants with diabetes and 40 without). Results showed evidence of a statistically significant difference in time to healing between the two groups (p<0.01) with median time to healing for those with diabetes being longer than for those without at 94 days (95% CI: 59, not estimable) compared to 78 days (95% CI: 53, 121).

Table 5 shows summary statistics for baseline immunological parameter concentrations by diabetic status. Mean concentration levels of ANG-2, IL-6, miR-126 and miR-146a were higher in those with diabetes than for those without. Within only those without diabetes (n=37) the mean IL-6 concentration (back transformed) at baseline was 6.4 pg/ml (SD 12.1) in those that went onto heal compared with 90 pg/ml (SD 243.1) in those who did not (p<0.01).

**Table 5.** Summary statistics for baseline immunological parameter concentrations, by diabetic status.

	Diabetic N=14	Non-diabetic N=37
*Angiopoietin-2 (pg/ml)*		
Mean (SD)	5760 (4908)	3123 (1351)
Median	4789	3111
Min, max	2063, 22195	1473, 7095
*Interlukein-6 ^†^ (pg/ml)*		
Mean (SD)	49.1 (91.2)	36.3 (146.2)
Median	11.6	4.4
Min, max	1.6, 300.4	1.6, 893.1
*miR-126 (AU/ml)*		
Mean (SD)	0.6 (0.6)	0.5 (0.3)
Median	0.4	0.5
Min, max	0.2, 2.7	0.1, 1.3
*miR-146a (AU/ml)*		
Mean (SD)	10.1 (6.8)	9.1 (7.0)
Median	9.3	7.3
Min, max	2.15, 25.5	1.1, 26.8

† 14 samples (13 healed; 1 unhealed) where concentration was below the assay detection limit of 3.1pg/ml with values set at 1.55 pg/ml (half the assay).

### Analyses of microbial species present in SWHSI

Table 6 shows summary statistics for baseline microbiological parameters. Overall, gastrointestinal bacteria (as defined above) were the most frequently observed (in 70.5%) followed by coliforms and skin commensals (both in 50.0%). Gastrointestinal bacteria were slightly less common in wounds that ultimately healed compared with those that did not (66.7% and 87.5% respectively). Coliforms were also more prevalent in wounds that did not heal (62.5%) compared with those that healed (47.2%). There were no clear differences between healers and non-healers in the prevalence of skin commensals or *Enterococcus* species. In general, proportions of other bacteria were higher for those who did not proceed to healing during follow up compared with those who healed. Fisher’s exact tests found no evidence of an association between healing status and gastrointestinal bacteria (p=0.40), coliform (p=0.70) or skin commensals (p=1.00).

**Table 6.** Baseline microbe presence, overall and by healing status.

	Overall	Not healed	Healed
	N=44	N=8	N=36
Gastrointestinal bacteria *, n (%)	31 (70.5)	7 (87.5)	24 (66.7)
**Coliform (one or more), n (%)**	22 (50.0)	5 (62.5)	17 (47.2)
**Skin commensals, n (%)**	22 (50.0)	4 (50.0)	18 (50.0)
**Enterococci, n (%)**	11 (25.0)	2 (25.0)	9 (25.0)
**More than one coliform, n (%)**	7 (15.9)	1 (12.5)	6 (16.7)
***Staphylococcus aureus*, n (%)**	8 (18.2)	2 (25.0)	6 (16.7)
**Anaerobe, n (%)**	6 (13.6)	0 (0.0)	6 (16.7)
***Pseudomonas aeruginosa*, n (%)**	3 (6.8)	2 (25.0)	1 (2.8)
**Group B streptococci, n (%)**	3 (6.8)	1 (12.5)	2 (5.6)
**No growth, n (%)**	3 (6.8)	1 (12.5)	2 (5.6)
**α haemolytic streptococci, n (%)**	1 (2.3)	0 (0.0)	1 (3.8)
**Candida, n (%)**	1 (2.3)	0 (0.0)	1 (2.8)
**Enterobacter cloacae, n (%)**	1 (2.3)	0 (0.0)	1 (2.8)
***Streptococcus milleri*, n (%)**	1 (2.3)	1 (12.5)	0 (0.0)

* Defined as the presence of one or more of “coliform”, *Pseudomonas*, *Enterococcus* or Enterobacter species.

There was no evidence of a statistically significant difference in time to healing (based on a log rank test, Table 7) dependent on presence of gastrointestinal bacteria (p=0.28), coliforms (p=0.42), skin commensals (p=0.77), *Enterococcus* species (p=0.98), more than one coliform (p=0.87), *Staphylococcus aureus* (p=0.89) or anaerobes (p=0.14).

**Table 7.** Time to wound healing, by microbe presence.

	n (%)		Median TTH * (95% CI)		Log rank
	Present	Not present	Present	Not present	p-value
**Gastrointestinal bacteria**	31 (70.5)	13 (29.5)	83 (53, 142)	70 (41, 112)	0.28
**Coliform (one or more)**	22 (50.0)	22 (50.0)	85 (48, 193)	76 (53, 122)	0.42
**Skin commensals**	22 (50.0)	22 (50.0)	70 (49, 94)	85 (53, 142)	0.77
**Enterococci**	11 (25.0)	33 (75.0)	76 (28, 233)	83 (53, 121)	0.98
**More than one coliform**	7 (15.9)	37 (84.1)	85 (16, 142)	78 (56, 121)	0.87
***Staphylococcus aureus***	7 (15.9)	37 (84.1)	78 (20, .]	78 (53, 121)	0.89
**Anaerobe**	6 (13.6)	38 (86.4)	70 (17, .]	78 (53, 142)	0.14

*Median TTH = median time to healing in days; . = unable to estimate

## Discussion

There has been limited previous analysis of immunological and microbial factors that predict surgical wound outcomes. This lack of research is matched by a lack of epidemiological research into SWHSI and consequently there is currently no published data (of which we are aware) that present even initial exploration of parameter heterogeneity and possible biomarkers for healing.

### Key results

We have provided important, initial descriptive data concerning some putative prognostic factors for healing in open surgical wounds. Most previous research exploring prognostic factors in wound healing has been cross-sectional ^27^ or in animals ^28^; however, we are uniquely able to explore potential associations between related baseline levels factors and verified healing outcomes because our study was nested in a larger inception cohort. This study provides metrics and descriptive statistics for this heterogeneous population of individuals with SWHSI and presents pilot data. Our study provides the first data set describing the extent of variability in the expression of circulating factors amongst individuals with SWHSI. This is vital information required for designing larger predictive biomarker discovery studies. It also indicates that it is feasible to nest smaller explorative biomarker studies within a larger cohort study within wound care. Notably, we found that at baseline individuals who did not heal during the follow up of the study displayed significantly higher plasma levels of IL-6 and ANG-2 in this study population. Interestingly, this trend was maintained for IL-6 even when we limited the analysis to people with no diabetes.

Experimental animal model studies of wound healing have suggested that IL-6 is essential for wound healing ^9, 10^, but conversely high IL-6 levels in exudates from venous ulcers have been associated with poor healing outcomes ^12^. This apparent discordance between human and animal studies is most likely due to differences between tractable experimental models and heterogeneous human cohorts, and also may be a result of distinct effects of IL-6 during the different stages of wound healing and repair.

Our results indicate a wealth of colonising or infecting microbial organisms present in SWHSI, including skin commensals, potential pathogenic microbes (e.g. *Staphylococcus aureus*, *Pseudomonas aeruginosa*), and coliforms. That such a high proportion of wounds had gastrointestinal bacteria is unsurprising given that 60% of wounds were of the abdomen or lower limb. Further studies can build on our results to determine the SWHSI microbiome at higher resolution using larger cohorts and unbiased (non-culture), sequence based techniques ^29, 30^. Interestingly, we did not observe any potential correlations between microbial species and healing outcomes of SWHSI. Larger studies are required to address this question. The results presented here provide a starting point and are essential in designing such studies. Understanding such potential correlations between bacterial colonisation, biomarkers, rational use of antibiotics, and healing outcomes is likely to have significant impact on wound care. 

### Limitations

This was a small study and therefore our comparisons of factors between people who healed and people who didn’t are likely to be underpowered to detect some differences as statistically significant. Furthermore, the small size of the cohort precluded extensive exploration of confounding factors between factor levels and wound healing, such as smoking status and infection status. However, diabetes was investigated as a potential confounding factor and simple stratified analyses were conducted. Caution should be taken when attempting to draw conclusions from such small cohorts and our findings require further validation in larger cohorts. Additionally, as the study concerns an inception cohort, the data are not designed to allow for causal links to be established, and whilst efforts have been made to reduce the risk of inflated Type I error, results would need to be verified in future research. Finally, we only collected data at baseline so we are unable to comment on the trajectories of factors over time.

### Implications

Overall, our study demonstrates the feasibility of biomarker discovery studies for SWHSI, despite the clinical heterogeneity of these complex wounds. Our results provide a solid foundation for designing robust biomarker discovery studies for SWHSI. Such studies are necessary for patient stratification, improved wound management, and are likely to have significant impact on healthcare resources and use of antibiotics. Given that therapeutic anti-IL-6 agents are now used in other areas of medicine, the potential correlation between enhanced baseline IL-6 levels and impaired healing is intriguing and requires further investigation in clinical and mechanistic studies.

We note that the relationships between possible biomarkers and healing is likely complex and could be mediated by other factors and/or cofounded by various cellular, wound and participant factors. Ultimately extensive further prognostic work in large cohorts of patients that adjust extensively for important factors are required. The design of such studies should be informed by careful exploration of existing data using systematic review approaches.

## Data availability

Access to data will require a formal data request form to be completed, as informed consent was not obtained from participants for public data sharing. The data request form can be obtained by contacting York Trials Unit at: catherine.arundel@york.ac.uk.

In accordance with the associated participant consent completed in relation to this study, anonymised sample data can be shared for use in medical research, where this research has been granted research ethics approval.

Access to data will be permitted on completion of a signed and authorised data request form. The recipient will be responsible for maintaining and protecting the integrity of the data and using this only for the purposes for which the request was made. Data must be stored securely and not passed on or have access granted to anyone other than those listed in the completed request form.
