# Supplementary material for: Characterisation of baseline microbiological and host factors in an inception cohort of people with surgical wounds healing by secondary intention reveals circulating IL-6 levels as a potential predictive biomarker of healing
Source: Wellcome Open Res. 2020 Nov 19;5:80. Originally published 2020 Apr 28. [Version 2] doi: 10.12688/wellcomeopenres.15688.2 (PMC8160585; doi:10.12688/wellcomeopenres.15688.2)
Supplement: Peer review history

# Peer review: Reviewer response for version 2

**DOI:** 10.21956/wellcomeopenres.18082.r43787

**Published:** 2021-05-27

**Authors:** Vincenzo Pappalardo

**Affiliations:** 1 Department of Surgery, Circolo ASST Settelaghi Hospital, Varese, Italy

The authors in this work offer an interesting analysis of the risk factors associated with the success or failure of secondary healing in surgical wounds. The work is well written and the subject is very interesting for both clinical and economic implications. 

 9 of 51 cases were identified as "non-healers", I think it is useful to clarify this concept by specifying early in the text what is meant by non-healers.

 It would also be useful to know whether techniques such as negative pressure, capable of modifying both the wound biome and the concentration of cytokines in the exudate, have been used or not.

Is the work clearly and accurately presented and does it cite the current literature?

Yes

If applicable, is the statistical analysis and its interpretation appropriate?

Yes

Are all the source data underlying the results available to ensure full reproducibility?

Yes

Is the study design appropriate and is the work technically sound?

Yes

Are the conclusions drawn adequately supported by the results?

Yes

Are sufficient details of methods and analysis provided to allow replication by others?

Yes

Reviewer Expertise:

Surgery.

I confirm that I have read this submission and believe that I have an appropriate level of expertise to confirm that it is of an acceptable scientific standard.

## References

[1] : Negative Pressure Therapy (NPWT) for Management of Surgical Wounds: Effects on Wound Healing and Analysis of Devices Evolution. *Surg Technol Int*.2019;34:56-67 31034574

# Peer review: Reviewer response for version 1

**DOI:** 10.21956/wellcomeopenres.17193.r40359

**Published:** 2020-11-05

**Authors:** Afshin Mosahebi

**Affiliations:** 1 UCL Division of Surgery and Interventional Science, Department of Plastic and Reconstructive Surgery, Royal Free Hospital, London, UK

Surgical wounds are important cause of morbidity, understanding & predicting wounds that will cause delay in healing will be an important step towards reducing this. This is an interesting study looking at biomarkers in would healing.

 Although it was a "fishing" exercise to look at a variety of markers, I think it is still useful to see elevated IL-6.

 Also, interesting to see the negative findings that no correlation between microbiological organisms & time to healing.

Is the work clearly and accurately presented and does it cite the current literature?

Yes

If applicable, is the statistical analysis and its interpretation appropriate?

Yes

Are all the source data underlying the results available to ensure full reproducibility?

Yes

Is the study design appropriate and is the work technically sound?

Yes

Are the conclusions drawn adequately supported by the results?

Yes

Are sufficient details of methods and analysis provided to allow replication by others?

Yes

Reviewer Expertise:

NA

I confirm that I have read this submission and believe that I have an appropriate level of expertise to confirm that it is of an acceptable scientific standard.

# Response

**Published:** 2020-11-05

**Authors:** Hannah Buckley

**Affiliations:** University of Leeds, UK

We thank the Reviewer for their time and expertise.  As the Reviewer states, this work was exploratory and as such we were cautious not to overstate the significance of our finding. We are glad that the Reviewer feels it is still useful to see elevated IL-6 and, as stated in our manuscript, believe our study supports that IL-6 merits further investigation.

# Peer review: Reviewer response for version 1

**DOI:** 10.21956/wellcomeopenres.17193.r39367

**Published:** 2020-07-22

**Authors:** Henry Hsia, Biraja Dash

**Affiliations:** 1 Division of Plastic and Reconstructive Surgery, Department of Surgery, Yale School of Medicine, Yale University, New Haven, CT, USA

This is a clearly written and interesting study that has the important aim of identifying potential biomarkers and prognostic factors for the success of secondary healing in surgical wounds. The authors are appropriately cautious in qualifying the work as a feasibility study with an “initial characterization” of a small cohort taken from a larger study previously published (Chetter 2019) ^1^ and are generally forthright about the limitations of the study. Table 1 provides clear inclusion and exclusion criteria for the 2018 study which had 393 participants of whom 54 provided consent for the current study. 

 The authors report that of 51 participants who provided blood samples, 42 were identified as healers and 9 as non-healers and that the non-healers had a significantly higher baseline level of IL-6 that was more than double that of the healers. While this is an interesting result, is it possible that the high level of IL-6 in these non-healing patients reflects the presence of a baseline high-risk clinical milieu? In addition to the caution provided by the authors regarding the limited numbers of participants, I would note the authors’ discussion from their 2019 study from which the current study’s participants were drawn: “Several factors… associated with a detrimental effect on healing have been identified including infection of a surgical wound healing by secondary intention at any point, wound area at baseline, high level of surgical contamination and reason for the surgical wound healing by secondary intention (e.g. inability to approximate the wound edges).”

 The high level of IL-6 (and other potential biomarkers) in these non-healing patients may simply reflect the presence of readily apparent clinical factors described by the authors in their previous study (e.g. infection, large wound area) that would not require a specific blood test to identify for prognostic purposes. The clinical utility of such blood tests as predictive biomarkers would be enhanced if they can be demonstrated in patients without any obvious clinical signs. Therefore, any suggestion that anti-IL-6 agents may have therapeutic utility in wound healing based on the study’s data (as the current manuscript does) is at best extremely premature.

 Regarding the study’s microbiological analyses, it is now generally recognized that for the purposes the authors stated in trying to identify potential prognostic factors, the use of sequencing-based methods is superior to the traditional culture-based methods employed by the authors (Gupta 2019) ^2^.

Is the work clearly and accurately presented and does it cite the current literature?

Yes

If applicable, is the statistical analysis and its interpretation appropriate?

Yes

Are all the source data underlying the results available to ensure full reproducibility?

Yes

Is the study design appropriate and is the work technically sound?

Partly

Are the conclusions drawn adequately supported by the results?

Partly

Are sufficient details of methods and analysis provided to allow replication by others?

Yes

Reviewer Expertise:

Wound healing.

We confirm that we have read this submission and believe that we have an appropriate level of expertise to confirm that it is of an acceptable scientific standard, however we have significant reservations, as outlined above.

## References

[1] : Patients with surgical wounds healing by secondary intention: A prospective, cohort study. *Int J Nurs Stud*.2019;89: 10.1016/j.ijnurstu.2018.09.011 62-71 10.1016/j.ijnurstu.2018.09.011 30343210

[2] : Amplicon sequencing provides more accurate microbiome information in healthy children compared to culturing. *Commun Biol*.2019;2: 10.1038/s42003-019-0540-1 291 10.1038/s42003-019-0540-1 31396571PMC6683184

# Response

**Published:** 2020-11-05

**Authors:** Hannah Buckley

**Affiliations:** University of Leeds, UK

We thank the Reviewers for their helpful suggestions. 

 We agree with the Reviewers that the high IL-6 levels may simply reflect other underlying factors/conditions associated with poor healing. We highlighted diabetes as one of those, noting the connection of IL-6 with diabetes but also that the observed trend in IL-6 levels was maintained even when we limited our analysis to people with no diabetes. We were cautious not to overstate the significance of our findings (i.e. we do not state that IL-6 levels are an independent prognostic biomarker). However, our study does support that IL-6 merits further investigation as a biomarker of healing in SWHSI, in future studies appropriately powered to also include a sufficient number of patients without specific confounding conditions (e.g. infection, diabetes), as suggested by the Reviewers.

 Our current study does not allow for this type of multiparametric analysis due to its small size. We accept that the suggestion of anti-IL-6 therapeutics is at this point speculative, but we believe that we were cautious not to suggest that our findings support imminent use of such therapeutics, rather they suggest that such therapeutic approaches can be potentially considered in the future.

 We also agree with the Reviewers with regards to the use of sequencing-based techniques for microbiological analyses. We had already made a mention of this "Further studies can build on our results to determine the SWHSI microbiome at higher resolution using larger cohorts and unbiased (non-culture) techniques 29”. We modify this sentence to: "Further studies can build on our results to determine the SWHSI microbiome at higher resolution using larger cohorts and unbiased (non-culture), sequence-based techniques” and also include the Gupta 2019 reference in addition to Misic et al. (ref 29).
